# Cumulative postoperative change in serum albumin levels and organ failure after living-donor liver transplantation: A retrospective cohort analysis

**DOI:** 10.1371/journal.pone.0285734

**Published:** 2023-05-11

**Authors:** Yoonjee Cho, Ja Eun Lee, Heejoon Jeong, Ji-Hye Kwon, Yu Jeong Bang, Gaabsoo Kim

**Affiliations:** Department of Anesthesiology and Pain Medicine, Samsung Medical Center, Sungkyunkwan University School of Medicine, Seoul, South Korea; Stanford University School of Medicine, UNITED STATES

## Abstract

Many studies have reported that hypoalbuminemia could be associated with organ failure after liver transplantation. However, most of them focused on serum albumin levels measured at specific time points and not on the trend of serum albumin change. We investigated whether a cumulative postoperative change in serum albumin level up to postoperative day (POD) 5 is related to organ failure in patients who underwent living-donor liver transplantation (LDLT). Data of adult recipients who underwent LDLT between January 2016 and December 2020 at a single tertiary hospital were reviewed (n = 399). After screening, three patients were excluded because of insufficient data. A cumulative change in serum albumin level was demonstrated using the area under the threshold (AUT, threshold = 3.0 g/dL) of the serum albumin curve up to POD 5. Based on the AUT, the patients were divided into a high-decrease group (n = 156) and a low-decrease group (n = 240). All analyses were conducted using 1:1 propensity score matching. The primary endpoint was the Sequential Organ Failure Assessment (SOFA) score on POD 5. The secondary endpoints were postoperative hospital stay and postoperative 90-day mortality. A total of 162 patients were included. The SOFA score on POD 5 was significantly higher in the High-decrease group compared with the Low-decrease group (5.2 ± 2.6 vs. 4.1 ± 2.3; mean difference: 1.1, 95% CI: 0.3 to 1.8; P = 0.005). However, the length of postoperative hospital stay (P = 0.661) and 90-day mortality (P = 0.497) did not differ between the groups. In conclusion, a cumulative postoperative change in serum albumin level up to POD 5 could help predict postoperative organ failure on POD 5 in patients who underwent LDLT.

## Introduction

Liver transplantation is a life-saving procedure for patients with end-stage liver disease [1]. Despite advances in medical therapy, liver transplantation has become the most effective treatment option for end-stage liver disease [2]. Recently, living-donor liver transplantation (LDLT) compared with deceased-donor liver transplantation, has offed a clear survival benefit to patients undergoing early transplantation [3]. LDLT can reduce the risk of worsening the recipient’s liver condition and result in better outcomes for the recipients because they are receiving a relatively healthy liver compared to deceased donor liver transplantation [4]. Moreover, improvements in surgical techniques and perioperative management have contributed to the recent widespread use of LDLT [4].

Albumin is the most common protein in human blood plasma and is important for maintaining oncotic pressure in plasma [5]. The serum albumin level is regarded as an important indicator of hepatic function [6], especially in patients with liver disease, because albumin is synthesized in the liver [7]. Patients undergoing liver transplantation commonly show low serum albumin levels owing to malnutrition and decreased hepatic function [8]. Therefore, several previous studies have investigated whether perioperative hypoalbuminemia is associated with worse outcomes such as organ failure and mortality in patients undergoing liver transplantation [9–11]. However, whether hypoalbuminemia should be treated, or the appropriate timing and volume of albumin supplementation remain controversial [11, 12]. Most previous studies have focused on serum albumin levels, which were measured once postoperatively, not on the cumulative change in serum albumin level [9, 11]. Moreover, they included living- and deceased-donor liver transplantation together in the analysis, despite the differences in surgical techniques and major characteristics of recipients.

Therefore, they were likely to have misunderstood the potential association between serum albumin levels and outcomes after liver transplantation. To overcome the limitations of previous studies, we investigated the association between the cumulative postoperative change in serum albumin up to postoperative day (POD) 5 and multiple organ failure only for recipients of LDLT. We hypothesized that a cumulative postoperative change in serum albumin until POD 5 was associated with the degree of multiple organ failure on POD 5 measured by the Sequential Organ Failure Assessment (SOFA) score.

## Material and methods

### Study design and ethical statements

The present study was a retrospective analysis of the medical records of all patients who received living-donor liver transplantation at a single tertiary medical center in South Korea. The study was approved by the Institutional Review Board of the Samsung Medical Center (approval no: SMC 2021-04-012-001; date of approval: April 6, 2021). The Institutional Review Board waived the need for written informed consent from participants due to the non-interventional design of the retrospective study. We adhered to the Strengthening the Reporting of Observational Studies in Epidemiology checklist for reporting this study [13]. All methods were performed in accordance with the ethical principles of the 1964 Declaration of Helsinki and its later amendments and were carried out following the approved guidelines.

### Study population

We reviewed the electronic medical records of all patients who underwent LDLT between January 1, 2016, and December 31, 2020. A total of 399 patients underwent LDLT at our institute and their medical records were assessed for eligibility. After screening, the patients were divided into a high-decrease group and a low-decrease group based on their cumulative postoperative change in serum albumin levels after the end of surgery until POD 5.

The patient groups were defined as follows:

### High-decrease group

Patients with a larger area under the threshold than that of the average of all patients in the serum albumin curve (greater reduction in serum albumin level over the average).

### Low-decrease group

Patients with a smaller area under the threshold than that of the average of all patients in the serum albumin curve (less reduction in serum albumin level compared to the average).

### Data collection and preparation

We obtained the baseline characteristics, perioperative course, and laboratory data from the electronic medical record system and clinical data warehouse of Samsung Medical Center. Baseline characteristics included age, sex, body mass index, Model for End-stage Liver Disease (MELD) score, comorbidities, and preoperative laboratory profiles including hemoglobin level, platelet count, prothrombin time, serum albumin, serum creatinine, total bilirubin, and sodium electrolyte levels. Intraoperative data, such as duration of surgery, ischemic duration of graft, duration of anhepatic phase, intraoperative volume of crystalloid, urine output, and estimated blood loss, were also collected. Clinical information about donor characteristics including graft-to-recipient weight ratio, graft type, and age of donor was also assessed. Data on daily serum albumin levels until POD5, SOFA scores on POD 5, human albumin supplementation until POD 5, length of postoperative hospital stay, postoperative 90-day mortality, Clavien-Dindo classification on POD 30, postoperative complications including hepatic artery thrombosis and portal vein thrombosis, biliary stenosis, biliary leakage, cytomegalovirus infection, and proteinuria up to POD 5 were collected for analysis.

**Fig 1:**
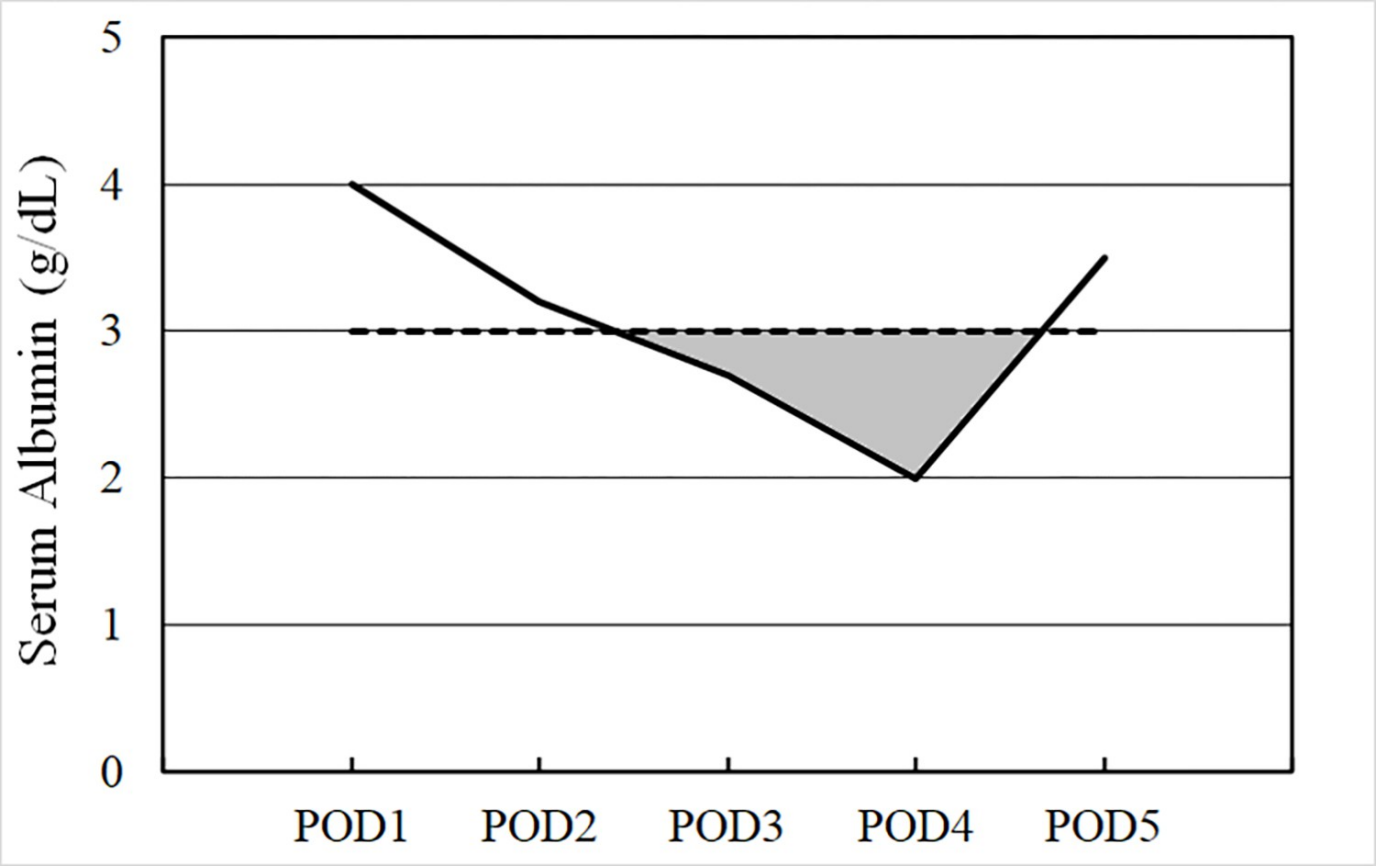
Example of calculating the area under the threshold of the serum albumin curve up to postoperative day 5 (preset threshold = 3.0 g/dL). The gray zone represents the area under the threshold. POD = postoperative day.

Daily serum albumin levels up to POD 5 were identified for each patient. After drawing a line graph of serum albumin levels from POD 1 to 5, the total area under the preset threshold (3.0 g/dL) was calculated using R version 4.2.1 (R Foundation for Statistical Computing, Vienna, Austria). The area under the threshold was considered a cumulative postoperative change in the serum albumin level. Fig 1 shows a representative area under the threshold calculation.

### Study outcomes and measurements

The primary endpoint was the association between the cumulative postoperative change in serum albumin levels and the degree of organ failure after LDLT. A cumulative change in serum albumin level was demonstrated using the area under the threshold. Organ failure was estimated using the SOFA score on POD 5. The secondary endpoints included the length of hospital stay after LDLT, postoperative 90-day mortality, and an association between the area under the threshold of the serum albumin curve and SOFA score on POD 5.

### Statistical analysis

Continuous variables are summarized as means (standard deviation) or medians [interquartile ranges], and categorical variables are summarized as frequencies (%). Absolute standardized differences in baseline characteristics and perioperative data were calculated to estimate possible imbalances between groups. Using imbalanced variables that showed an absolute standardized difference > 0.2, a 1:1 propensity score-matched analysis was performed using the nearest-neighbor method with a caliper width of 0.2 in a pairwise manner. The Wilcoxon rank-sum test was performed to determine differences in continuous variables, and Fisher’s exact test was used to compare a categorical variable. The confidence intervals were calculated using the Hodges-Lehmann estimator. The association between the area under the threshold of the serum albumin curve until POD 5 and the SOFA score on POD 5 was assessed using Spearman’s correlation analysis.

The value of P < 0.05, two-sided, was considered statistically significant. SPSS version 24 (IBM., Armonk, NY, USA) and R version 4.2.1. was used for the analysis. *Priori* statistical power calculation was not performed in this observational cohort analysis. Therefore, the sample size in this study was determined using the available dataset.

## Results

**Fig 2:**
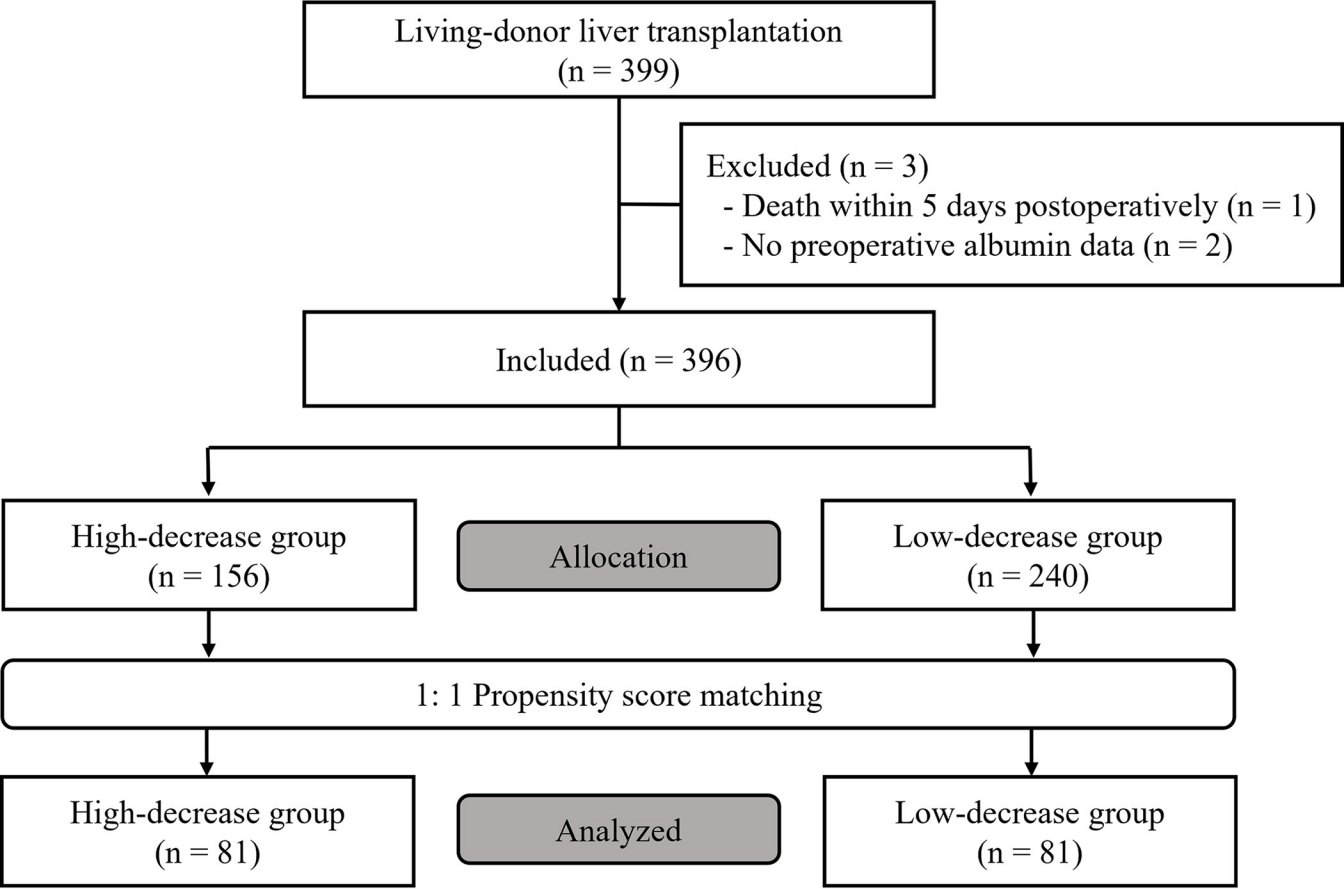
Flow diagram of the study.

A total of 399 patients underwent LDLT at our institute between January 2016 and December 2020. After screening their medical records, three patients were excluded because of insufficient data: death within five days postoperatively (n = 1) and no preoperative serum albumin data (n = 2). Therefore, a total of 396 patients were included in the analysis. After 1:1 propensity score matching, 162 patients (81 in each group) were analyzed (Fig 2). The patient demographics and perioperative data are shown in Table 1.

**Table 1:** Baseline characteristics of patients.

Variable	Before Propensity Score Matching			After Propensity Score Matching		
	High-decrease group (n = 156)	Low-decrease group (n = 240)	ASD	High-decrease group (n = 81)	Low-decrease group (n = 81)	ASD
*Baseline demographics*						
Age, year	57 [52–61]	56 [52–61]	0.05	57 [51–60]	57 [52–61]	
Sex, male	108 (69)	182 (76)	0.15	58 (72)	48 (59)	
Body mass index, kg/m^2^	23.7 [21.8–25.9]	24.7[23.0–27.1]	0.26	23.6 [22.1–27.2]	23.6 [21.8–26.4]	0.09
MELD score	17 [13–25]	8 [7–13]	0.93	15 [12–25]	14 [10–20]	0.21
Hypertension	13 (8)	32 (13)	0.16	10 (12)	9 (11)	
Diabetes mellitus	24 (15)	42 (18)	0.06	14 (17)	18 (22)	
*Preoperative laboratory profile*						
Hemoglobin, g/dL	9.3 [8.2–11.1]	12.6 [10.6–14.0]	1.18	9.8 [8.3–11.6]	10.1 [8.6–11.6]	0.11
Platelet count, x 10^3^/μL	58 [40–82]	79 [51–121]	0.44	56 [38–81]	60 [45–92]	0.01
Prothrombin time, INR	1.5 [1.3–1.9]	1.2 [1.1–1.3]	0.53	1.5 [1.3–1.8]	1.4 [1.2–1.7]	0.14
Serum albumin, g/dL	3.0 [2.7–3.2]	3.7 [3.2–4.0]	1.33	3.0 [2.8–3.4]	3.1 [2.8–3.4]	0.16
Serum creatinine, mg/dL	0.76 [0.59–1.02]	0.77 [0.62–0.90]	0.26	0.75 [0.60–1.01]	0.74 [0.56–0.98]	0.06
Total bilirubin, mg/dL	2.8 [1.6–6.9]	1.0 [0.6–1.7]	0.47	2.6 [1.3–8.2]	1.9[1.1–4.9]	0.14
Sodium, mmol/L	136 [132–139]	141 [139–142]	0.97	138 [136–141]	139 [137–142]	0.07
*Donor characteristics*						
GRWR	1.13 [0.91–1.32]	1.08 [0.95–1.29]	0.16	1.17 [0.89–1.34]	1.12 [1.00–1.39]	0.21
Graft type, right lobe	142 (91)	227 (95)	0.14	73 (90)	77 (95)	0.19
Age of donor, year	33 [24–44]	34 [25–48]	0.13	33 [24–44]	34 [24–44]	0.01
*Intraoperative data*						
Duration of surgery, min	416 [364–477]	419 [366–493]	0.10	413 [369–458]	409 [352–477]	
Cold ischemic time, min	82 [66–101]	82 [68–98]	0.11	81 [65–103]	83 [69–102]	
Warm ischemic time, min	36 [31–47]	37 [33–53]	0.34	38 [32–52]	36 [31–54]	
Anhepatic time, min	101 [89–122]	99 [80–126]	0.13	102 [93–123]	103 [80–140]	
Crystalloid infusion, mL	5400 [3900–6800]	4600 [3600–6000]	0.26	5000 [390–6700]	4600 [3800–6000]	0.01
Urine output, mL	530 [390–780]	980 [630–1440]	0.91	520 [400–850]	600 [430–940]	0.06
Estimated blood loss, mL	300 [170–500]	180 [100–300]	0.55	240 [150–400]	250 [160–400]	0.01

Data are presented as median [interquartile range] or frequency (%). ASD, absolute standardized difference; MELD, Model for End-stage Liver Disease; INR, international normalized ratio; GRWR, graft to recipient weight ratio.

The SOFA score on POD 5 was significantly higher in the high-decrease group than in the low-decrease group (mean ± SD; 5.2 ± 2.6 vs. 4.1 ± 2.3; mean difference: 1.1, 95% CI: 0.3 to 1.8; P = 0.005) (Table 2). There were no significant differences in the SOFA subscores except renal SOFA (0.5 ± 0.8 vs. 0.2 ± 0.6; mean difference: 0.2, 95% CI: -0.3 to -0.4; P = 0.025). The length of hospital stay after surgery (P = 0.822) and postoperative 90-day mortality (P = 0.497) did not differ between the groups (Table 2). Postoperative morbidities were also presented in Table 2.

**Table 2:** Postoperative outcomes after living-donor liver transplantation after propensity score matching.

Variable	High-decrease group (n = 81)	Low-decrease group (n = 81)	Mean difference (95% CI)	P value
Sequential Organ Failure Assessment score on POD 5	5.2 (2.6)	4.1 (2.3)	1.1 (0.3 to 1.8)	0.005
Respiratory SOFA	0.8 (1.0)	0.6 (0.9)	0.2 (-0.1 to 0.5)	0.205
Coagulation SOFA	2.0 (0.9)	1.8 (0.9)	0.2 (-0.1 to 0.5)	0.088
Cardiovascular SOFA	0.1 (0.4)	0.2 (0.5)	-0.1 (-0.2 to 0.1)	0.467
Hepatic SOFA	1.3 (0.9)	1.1 (1.0)	0.2 (-0.1 to 0.5)	0.233
Neurological SOFA	0.4 (0.7)	0.2 (0.5)	0.1 (-0.1 to 0.3)	0.254
Renal SOFA	0.5 (0.8)	0.2 (0.6)	0.2 (-0.3 to -0.4)	0.025
Patients who received albumin supplementation until POD 5	81 (100)	76 (94)	NA	0.120
Length of postoperative hospital stays, day	30 (25)	29 (18)	1 (-5 to 8)	0.661
Postoperative 90-day mortality	2 (3)	0 (0)	NA	0.497
Clavien-Dindo classification grade ≥ 3 on POD 30	25 (31)	28 (35)	NA	0.738
Postoperative complications				
Hepatic artery stricture or thrombosis	4 (5)	1 (1)	NA	0.367
Portal vein stricture or thrombosis	5 (6)	3 (4)	NA	0.720
Biliary stenosis	40 (49)	32 (40)	NA	0.268
Biliary leakage	10 (12)	16 (20)	NA	0.199
CMV infection	3 (4)	4 (5)	NA	0.999
Proteinuria up to POD 5	47 (58)	45 (56)	NA	0.874

Data are presented as mean (standard deviation) or frequency (%). POD, postoperative day; SOFA, Sequential Organ Failure Assessment; CI, confidence interval; NA, not applicable; CMV, cytomegalovirus.

**Fig 3:**
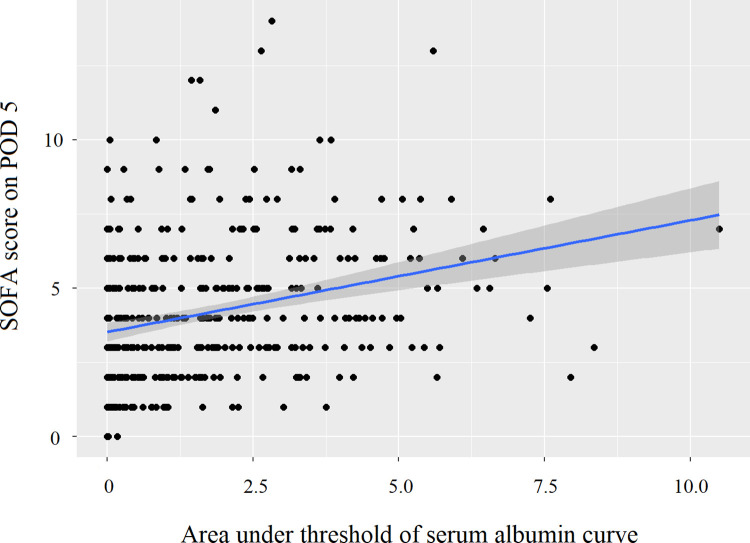
Correlation between the area under the threshold of the serum albumin curve up to POD 5 and the Sequential Organ Failure Assessment score on POD 5 (Spearman’s ρ = 0.32; P < 0.001). POD, postoperative day.

The area under the threshold of the serum albumin curve until POD 5 was significantly correlated with the SOFA score on POD 5 (Spearman’s ρ = 0.32, P < 0.001) (Fig 3).

## Discussion

In this retrospective analysis, patients with a low postoperative decrease in cumulative serum albumin level had a lower SOFA score on POD 5 than those with a high decrease after LDLT. A cumulative change in serum albumin level was demonstrated using the area under the threshold of the serum albumin curve up to POD 5. A significant association between the area under the threshold and the SOFA score on POD 5 was also observed.

Postoperative hypoalbuminemia is frequently observed in patients undergoing major abdominal surgery [14]. Particularly, regarding serum albumin levels as an important indicator of perioperative hepatic function in patients undergoing liver transplantation, many studies have focused on whether perioperative hypoalbuminemia is associated with worse outcomes or whether albumin supplementation could improve postoperative outcomes, such as organ failure and survival [10, 11, 15]. Recently, Hiroi *et al*. [9] reported that serum albumin levels might not influence cumulative organ function after liver transplantation n. However, they classified the patients into the low albumin group if their serum albumin levels fell below 3.0 g/dL even once during the first postoperative week, regardless of their average serum albumin level. Considering that hypoalbuminemia is common in recipients after liver transplantation, this classification might not be appropriate for accurately estimating the effect of serum albumin on postoperative outcomes. In our study, 94% (76/81) of the recipients in the low-decrease group had serum albumin levels below 3.0 g/dL at least once up to POD 5.

Therefore, we explored different methods that focus on the cumulative change in serum albumin levels. According to a previous study, a feasible method using the area under the threshold can provide comprehensive insight to assess variables that frequently change during the study period, such as blood pressure [16]. Considering that serum albumin levels also frequently change after LDLT, the postoperative course of serum albumin can be identified more clearly through the new approach. Moreover, the AUT method added up only the area below the threshold. Even if serum albumin level rose over the threshold after supplementation of human albumin, it was not counted toward the final AUT value. This is thought to have contributed to reducing the bias caused by albumin supplementation during the study period.

In the present study, low SOFA scores in patients with a lower reduction in serum albumin level postoperatively were most pronounced for the renal function subscore, which is in line with the previous finding of Sang et al. [10]. Although the precise mechanism of its positive effect on the renal system has not been fully elucidated, albumin has shown a renoprotective effect in many previous reports [17–19]. The explainable principles include the antioxidant effect against uremic toxins, preventing apoptosis of renal tubular cells, alleviating the nephrotoxic effects of several medications, and endothelial stabilization to improve renal blood flow [17, 20, 21]. These mechanisms would have worked similarly for the patients in this study.

The large area under the threshold of the serum albumin curve was associated with the high SOFA score. Our finding suggested that the new method using the area under the threshold could help predict the postoperative prognosis after LDLT. Therefore, we thought that a continuous increase in the area under the threshold of the serum albumin curve could be applied as a useful alarm indicating the possibility of organ failure progression. In this study, however, the reduction in SOFA scores did not show improvement in the postoperative outcomes, such as postoperative hospital stay and 90-day mortality. Because preoperative medical conditions and intraoperative management strongly influence postoperative outcomes, balancing these factors using propensity score matching may result in insignificant differences in postoperative outcomes. Thus, prospective further study is needed to determine whether the area under the threshold of the serum albumin curve is associated with postoperative morbidity and mortality after LDLT.

Our study had several limitations. First, this was a retrospective single-center study; therefore, our management protocols and strategies for LDLT may have influenced the results. Prospective multicenter research is needed to reduce biases and validate our results. Second, the SOFA score is a comprehensive scoring system to assess systemic multiorgan failure. Therefore, despite our effort to reduce the influence of the confounders, other comorbidities or medical conditions besides serum albumin level could have affected or even distorted the SOFA score. Third, although many previous reports have used a serum albumin level of 3.0 g/dL to define hypoalbuminemia after liver transplantation, it is not clear whether applying the same level for calculating the area under the threshold is appropriate. Finally, postoperative albumin supplementation during the study period could have affected the changes in serum albumin levels. Although albumin supplementation was determined by the same protocol in all patients and the frequency of albumin supplementation was not different between the two groups, postoperative albumin supplementation could have attenuated the reduction of serum albumin levels in patients with moderate to severe hypoalbuminemia.

## Conclusion

In conclusion, a cumulative postoperative change in serum albumin levels up to POD 5 could predict postoperative organ failure in patients who underwent LDLT. A prospective controlled trial is warranted to determine the feasibility of new measurement using the area under the threshold of the serum albumin curve.
